# Kretschmer´s Sensitive Delusion Of Reference: Clinical Case Report

**DOI:** 10.1192/j.eurpsy.2024.1550

**Published:** 2024-08-27

**Authors:** S. G. Rodrigues, J. L. Freixo, T. Novo, D. S. Brandão

**Affiliations:** Psychiatry, Unidade Local de Saúde do Alto Minho, Viana do Castelo, Portugal

## Abstract

**Introduction:**

The sensitive delusion of reference represents a clinical entity described by Kretschmer in 1918, arising in people with sensitive personality. This personality type is mainly characterized by a tendency towards social isolation, introversion, low self-esteem and by a greater sensibility to interpersonal judgement. In this personality type, the presence of specific environmental triggers may provoke a reference delusion of persecutory content, feelings of guilty and injustice. Although eliminated from the current diagnostic classifications, the sensitive delusion of reference represents a key milestone in the history of psychopathology.

**Objectives:**

The goal of this report is to report on a clinical case of a patient diagnosed with Kretschmer´s sensitive delusion of reference.

**Methods:**

The present work consists on a descriptive report of a clinical case through consultation of the patient´s clinical file as well as a survey of relevant articles on *Pubmed.*

**Results:**

This is a 38 year old, married man with no children. He describes himself as a private, introverted individual, who has little interaction with his peers, and has been very sensitive to criticism ever since his adolescence. His first psychiatry appointment took place in April 2021, following the medical referral of his general practitioner as, according to the patient´s mother and wife, he had been, for quite some time, implying that his mobile phone had been under wire and that someone had been monitoring him. As stated by these relatives, these ideas surfaced after a workplace conflict. At the time, the patient was medicated with olanzapine 10mg and lorazepam 2.5mg before bedtime, exhibiting significant improvements with full remission of psychotic symptomatology. Succeeding the antipsychotic tapering attempt, the patient had begun to suffer from insomnia and recrudescence of psycotic symptomatology, namely, the delusion ideation of persecutory content and auditory hallucinations, as a result, the previous treatment regimen was resumed, which resulted in significant improvements of the clinical picture. Following new observation, in 2023, the patient mentions weight gain and drowsiness during the day, leading to the switch of olanzapine 10mg for aripiprazole 15mg. The current treatment plan consists of aripiprazole 15mg once a day and lorazepam 1mg 1/2 before bedtime, resulting in an improvement of the previous complaints and maintenance of the psychosocial functioning, unaccompanied by psychotic symptomatology.

**Conclusions:**

In conclusion, and relatively to the condition´s prognosis, Kretschmer observed that, although in some situations the episodes were brief and self-limiting, in others, the patients maintained psycothic symptomatology, during the following years. In the present clinical case we recognised the need to sustain the antipsycothic treatment regimen, as the respective dosage reduction lead to an aggravation in symptomatology.

**Disclosure of Interest:**

None Declared

